# Long-term behavioral effects of social separation during early life in a social mammal, *Octodon degus*

**DOI:** 10.1038/s41598-023-36745-6

**Published:** 2023-06-12

**Authors:** Rina Ukyo, Akio Shinohara, Chihiro Koshimoto, Goro A. Nagura-Kato, Seiji Ieiri, Yasuhiro Tsuzuki, Shinsuke H. Sakamoto

**Affiliations:** 1 Interdisciplinary Graduate School of Agriculture and Engineering, University of Miyazaki, Miyazaki, Japan; 2 Division of Bio-Resources, Department of Biotechnology, Frontier Science Research Center, University of Miyazaki, 5200 Kihara, Kiyotake-cho, Miyazaki-shi, Miyazaki 889-1692 Japan; 3 Faculty of Agriculture, University of Miyazaki, Miyazaki, 889-2192 Japan; 4 Center for Animal Disease Control, University of Miyazaki, Miyazaki, Japan

**Keywords:** Animal behaviour, Developmental disorders, Stress and resilience, Neonatal brain damage, Psychology, Psychiatric disorders, Anxiety

## Abstract

Social separation is thought to induce a strong stress response in social juvenile mammals, but little is known about how this response might vary throughout the development. The present study examines the long-term effects of early-life stress (ELS) induced by social separation on individual behaviors later in life using the social and precocious species *Octodon degus*. Four experimental groups were established a positive control group of mothers and siblings from six litters comprised the socially housed (SH) group, while pups from seven litters were randomly assigned to three treatments: pups experiencing no separation (NS) treatment while their siblings did; repeated bouts of consecutive separation (CS); intermittent separation (IS). We analyzed the effects of separation treatment on the frequency and duration of freezing, rearing and grooming behaviors. ELS was correlated with higher hyperactivity, and hyperactivity increased with more frequent separation. However, the behavioral trend of the NS group changed to hyperactive in long-term observation. The findings suggest that the NS group was indirectly affected by ELS. In addition, suggesting ELS acts to converge an individual’s behavioral tendencies in a certain direction.

## Introduction

It is widely accepted that early-life environmental stresses can increase the likelihood of developing mental illness later in life. During the process of brain development, synapse formation begins at birth and continues for several months postnatally. This period is crucial for the formation of efficient neural networks in the brain^1^. Thus, animals are highly sensitive to their environment, especially at the time of birth, and need a stable environment to thrive^2^. Stress in animal pups after birth has been shown to interfere with normal behavioral and physiological development and to be an important risk factor for post-developmental mental health^3–5^. Reportedly, chronic stress in animals can lead to changes in their endocrine levels^6^. Neuroscience and neuroanatomy studies have documented how negative mental health experiences adversely affect the development of limbic circuit functions and structures^7,8^. The effects of stress also vary by sex because, during an animal’s development, sex differences induced by gonadal hormones influence brain development and hormonal dynamics^9–12^.

The most significant form of early-life stress (ELS) occurs during social separation, which involves the interruption of direct communication and contact between an animal and its caregiver of the same group, such as a mother, regardless of the animal's will^13^. Social animals are affected by social separation and experience significant stress, which leads to detrimental psychological and behavioral effects^14–17^. Thus, social separation during this sensitive period has a crucial impact on juvenile development. However, there are only few studies with long-term observations. Hence, the sex-dependent effects of separation and indirect effects due to separations of siblings are understudied because these effects are expected to become apparent after a long time.

The effects of social separation may cause problems in various animal breeding situations^18,19^. For example, on farms, livestock production frequently leads to young juveniles being separated, and in zoos, social separation of offspring is often unavoidable due to a limited number of animals in captivity, abandonment, or inter-individual conflict^20,21^. These ELS events induced by separation are possible causes of serious issues in the juvenile’s developmental process. However, to research the effects of separation, tracking an individual’s development in a controlled situation, including the effects of social environmental factors on livestock or zoo animals, is challenging because of problems in production, long experimental periods, need for large sample sizes, space constraints, and high costs. Hence, research focusing on separation experiments using model mammals is urgently needed.

**Figure 1:**
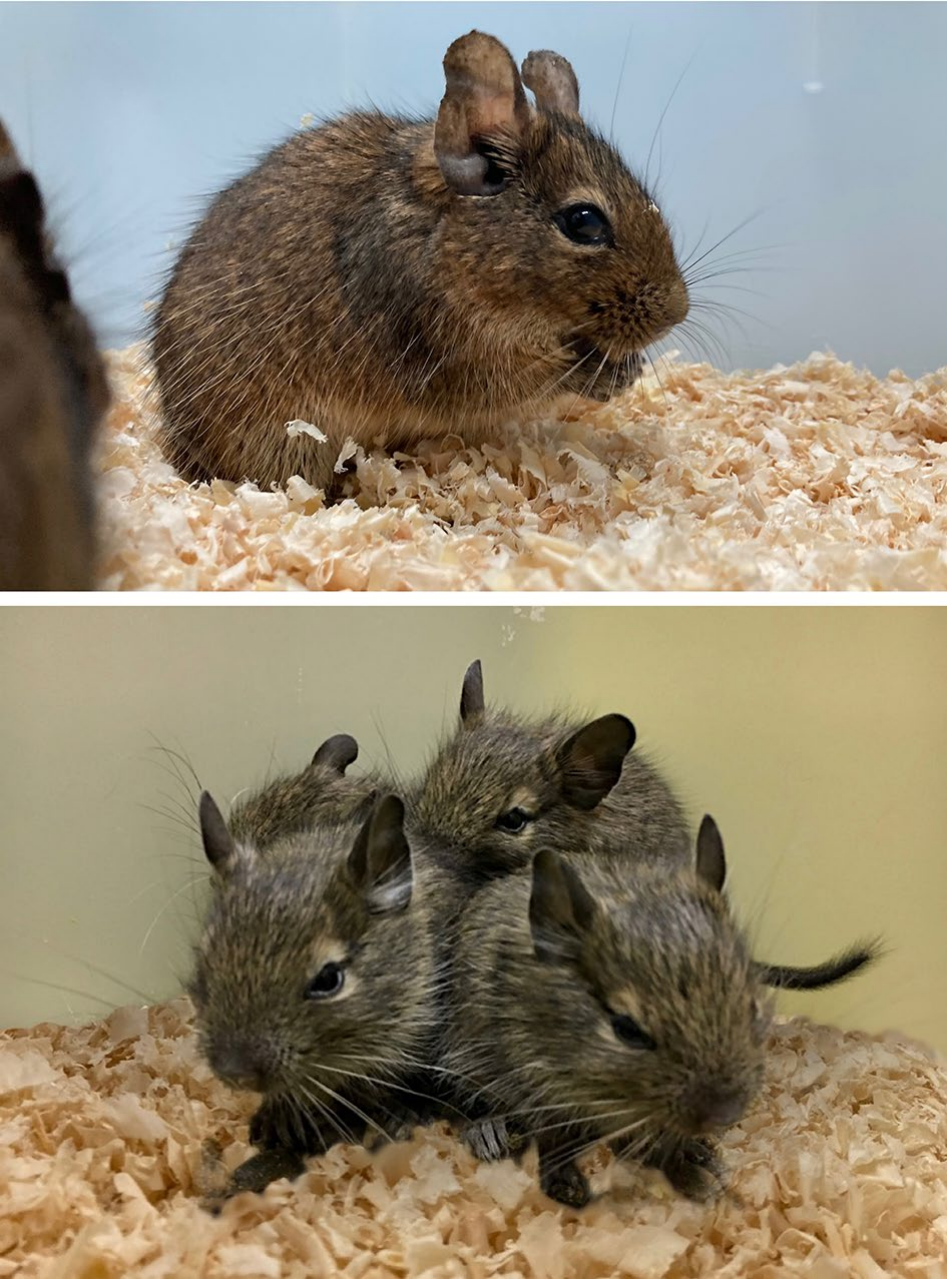
*Octodon degus*. The upper photo shows an adult and the lower one shows pre-weaned pups.

*Octodon degus* (Fig. 1) are highly intelligent, diurnal, herbivorous, and small, social mammals^22–24^. They live in groups^25^ and have complex vocal communication^8,26^. Their groups normally comprise 1–3 males and 1–10 females and have a promiscuous mating system^23^. *Degus* nest and raise their young communally, with females nursing not only their own but also other females’ young born at the same time^27^. The gestation period is about 90 days and natural weaning occurs at 4–6 weeks. Furthermore, they are precocious, meaning they are considered advanced at birth because they can move independently and vocalize within 3–4 h of birth, display functional grooming (face washing, hind-paw scratching, and rapid headshake) by the 1st or 2nd day and communicate with their parents and siblings^28^. Thus, as a model species, we can analyze behavioral reactions and psychological factors at an advanced developmental level compared to experimental mice or rats. In addition, this species migrates on the ground during the day and regulates foraging behavior by emitting vocalizations while maintaining visual contact^26,27,29,30^; isolated juveniles emit 'whistle' distressed vocalizations (DVs) to induce retrieval by adults^26,31^; specific calls are emitted upon reunion, with lactating females emitting 'mothering calls'; it has been reported that emitting characteristic lactation calls induces suckling behavior and regulates lactation^32^. This species thus demonstrates strong social attachment and strong stress responses to short time separation from the caregivers^17,33^. Since diurnal rodent models are rare, studying *degus* is important for translating the laboratory findings to other diurnal animals and humans^34^. Because of these characteristics, *degus* are considered to be a useful model animal in the fields of psychopathology, psychology, neuroscience, and animal behavior^35^.

In previous research, the focus was on the development of the brain, hormones, and endocrine stimulators such as neurotransmitters. Studies have reported an increase in stress hormones in the plasma following separation, and a daily 24-h separation increases individual activity^33^. In addition, ELS results in a widespread reduction in brain activity in the infant’s brain, changes in interregional functional coupling, and can lead to attention-deficit and brain metabolic hypoactivity in degus^36,37^. Nevertheless, these results were primarily observed around the weaning time (Postnatal day 35, PND35), and studies investigating how this response varies through the developmental process have only been reported by Rivera^38^. In addition, to our knowledge, there have been no studies that illustrate sex -dependent responses to direct ELS (DELS) or to directly examine the behavioral effects of indirect ELS (IELS) in social animals.

**Figure 2:**
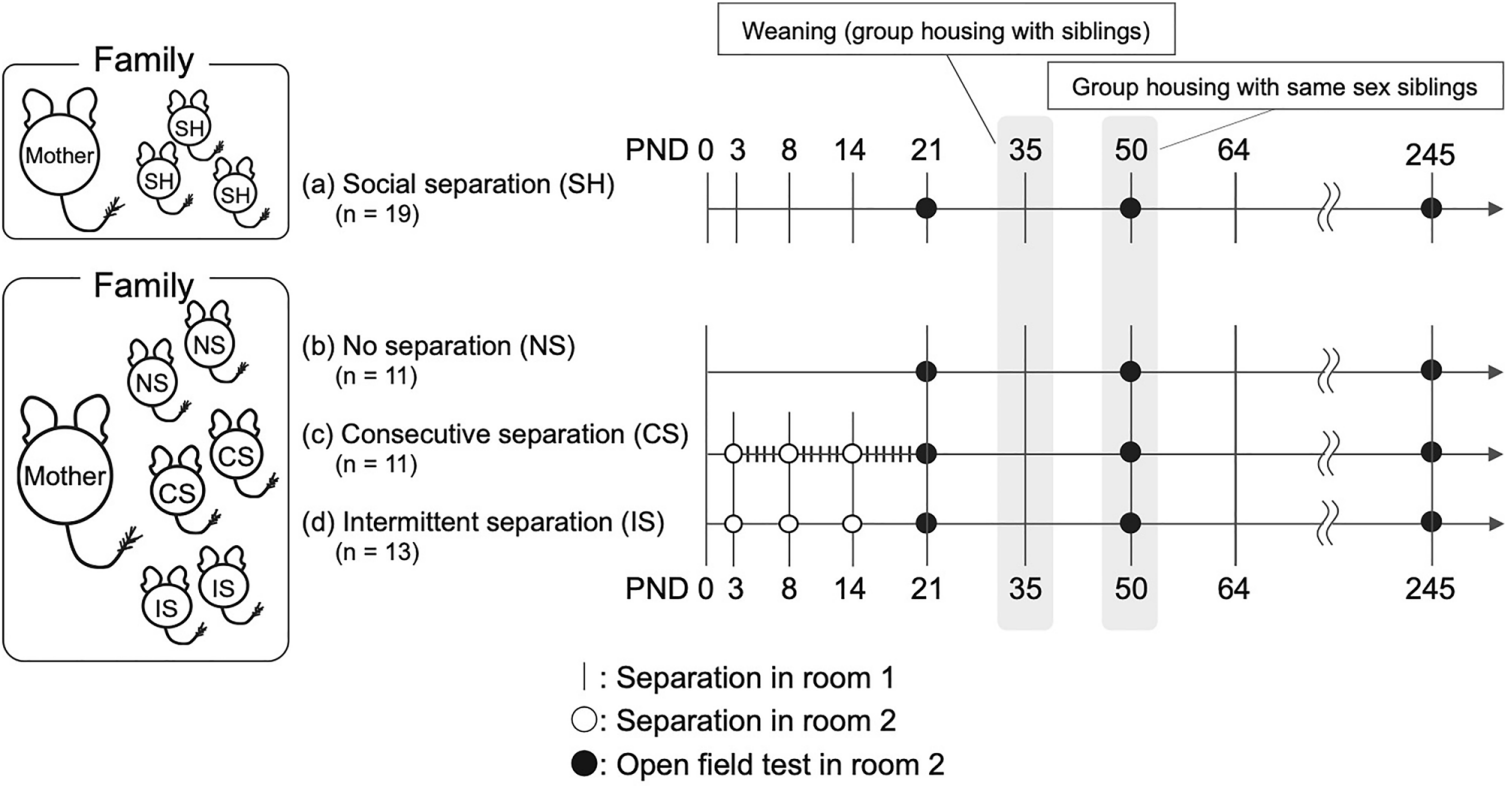
Experimental scheme of the separation stress treatments. (**a**) Social housing group (SH): the animals were reared undisturbed with their family in the home cage from PND0 to PND20. On PND21, the degu pups underwent the OFT in room 2 where they could not communicate with their mother using olfactory, acoustic, visual, or social contact (touching) signals. After behavioral testing, the animals were returned to their home cage and reared with their mother and siblings until PND35 (the day of weaning). On PND35, the degu pups were artificially weaned by removing the mother from the home cage. From PND35 to PND50, the siblings were housed in the same cage and, after PND50, same-sex siblings were reared in one cage. (**b**) No separation group (NS): the animals were reared undisturbed with their mother in the home cage from PND0 to PND20. Manipulations after PND21 were the same as those in the SH group. (**c**) Consecutive separation group (CS): from PND3 to PND20, the degu pups were removed from their mother and home cage and isolated for 1-h daily. After this separation, pups were returned to their family and home cage and left undisturbed until the next day. Note that on PND3, PND8, and PND14, separation was performed in room 2, while on PND4–7, PND9–13, and PND15–20 separation was performed in room 1. Manipulations after PND21 are the same as those in the SH and NS groups. (**d**) Intermittent separation group (IS): on PND3, PND8, and PND14, the degu pups were separated from their mother and home cage and isolated for 1-h daily in room 2. After this separation, pups were returned to their family and home cage and left undisturbed until the next separation day. Manipulations after PND21 are the same as those in the other three groups.

Thus, to reveal the long-lasting effects of ELS experienced from PND3 to PND20 on individual behaviors exhibited after development, we used the open-field test (OFT) and compared behaviors with respect to PND21, PND50, and PND245 among the four following groups: (i) social housing (SH) group (control group): the whole litter did not experience any separation treatment; (ii) no separation (NS) group: the pups did not experience separation from their mother, but their siblings did; (iii) consecutive separation (CS) group: the pups were removed from their mother and home cage for 1-h every day; and (iv) intermittent separation (IS) group: the pups were removed from their mother and home cage for 1-h on PND3, PND8, and PND14 (see Supplementary Table S1 and Fig. 2). We defined the NS group as the IELS group and the CS and IS groups as the DELS group. Furthermore, sex differences were analyzed among these groups.

## Results

### Effects of ELS on bodyweight gaining

**Figure 3:**
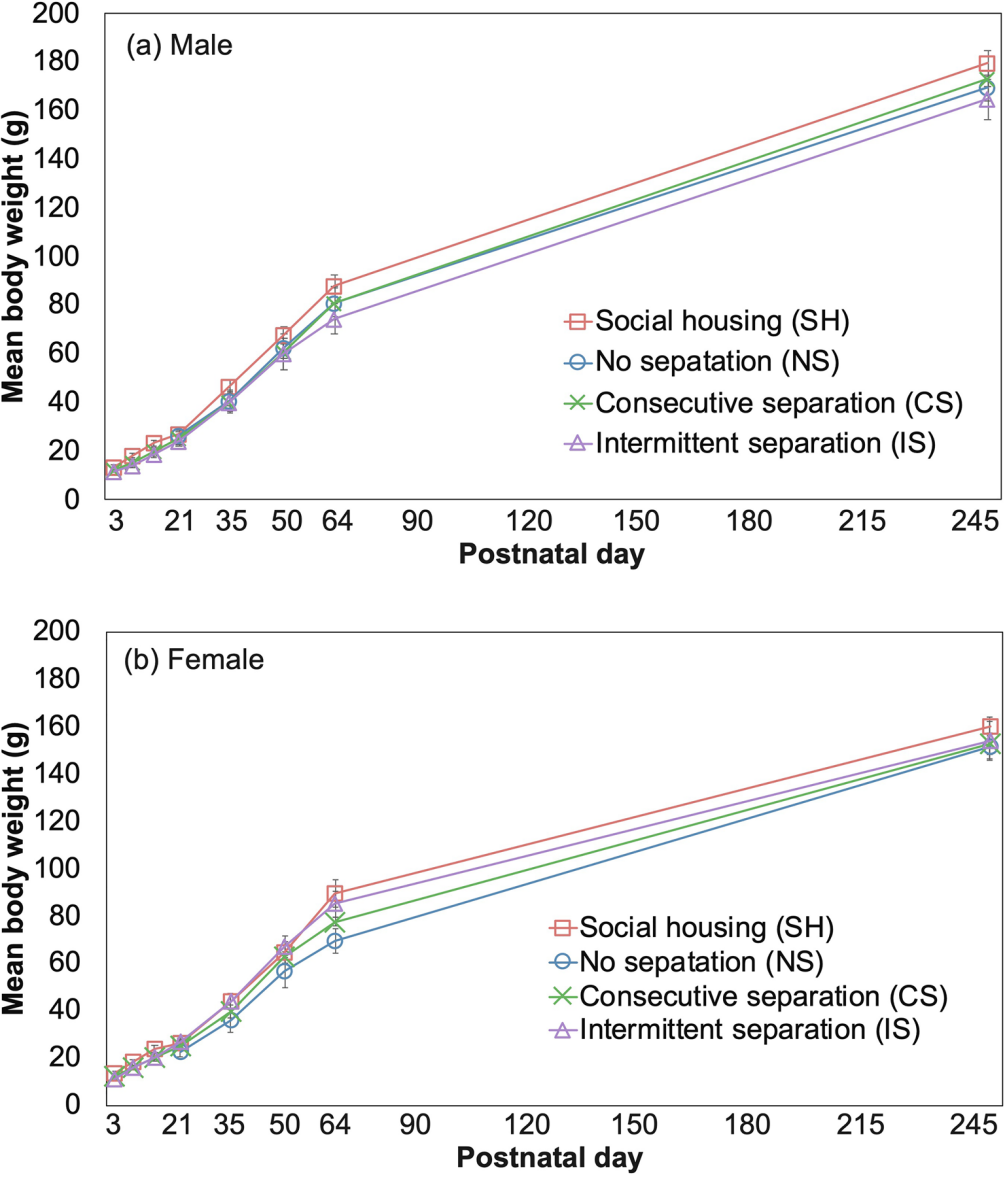
Mean weight change in (**a**) male and (**b**) female for four separation groups, the social housing (SH), no separation (NS), consecutive separation (CS), and intermittent separation (IS) of each PND. Error bars indicate standard error. In statistical analysis, the data from PND21 onwards, when data for all individuals were available.

Males gained more weight than females (Fig. 3, Supplementary Table S2; PND × Sex). There were no obvious effects of ELS on the bodyweight gain (Supplementary Table S2; Separation group), and no significant relationship was detected between the stress treatment and sex factors (Fig. 3, Supplementary Table S2; Group × Sex).

### Effects of ELS and PND on behaviors

**Figure 4:**
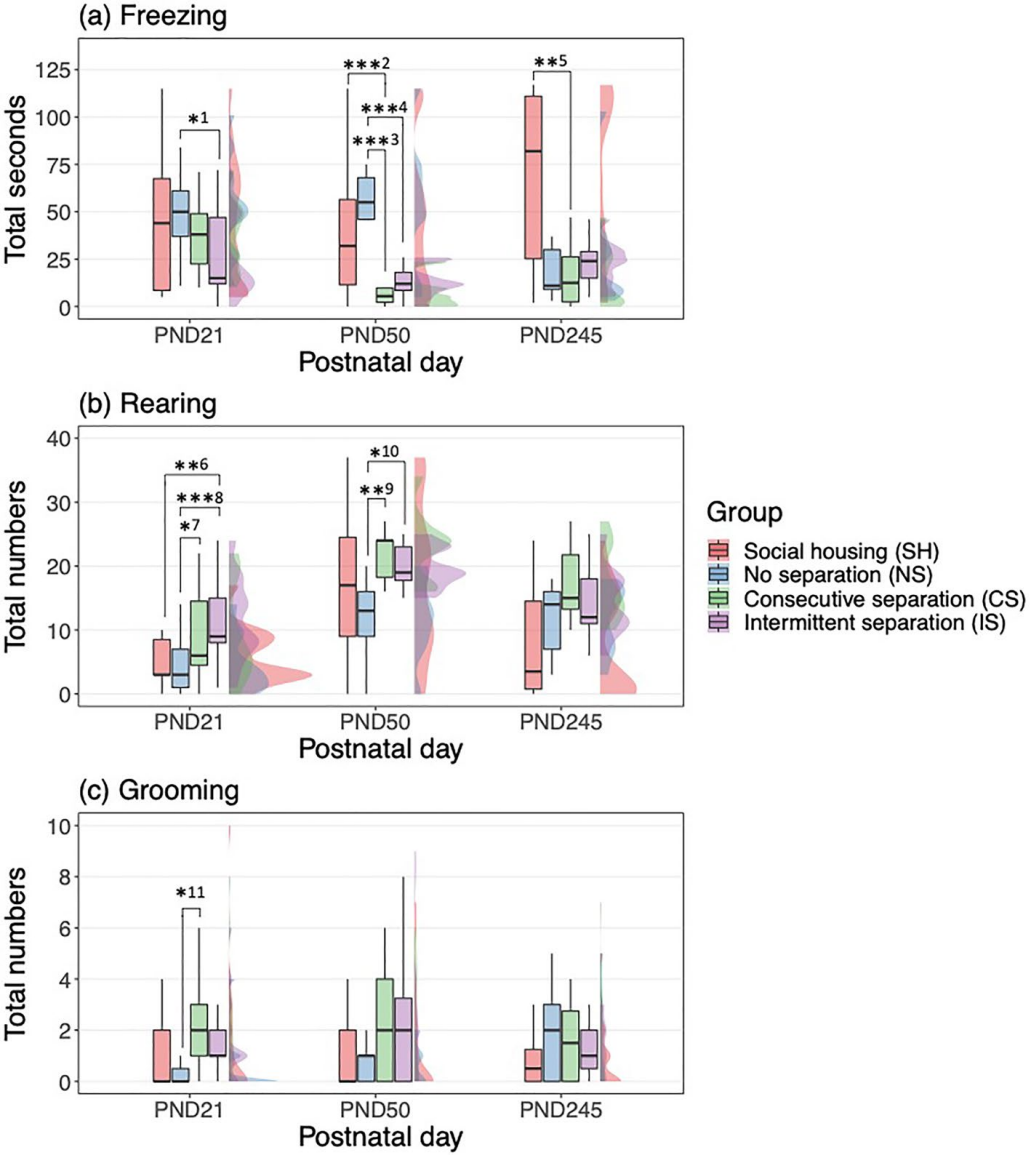
(**a**) Total time spent freezing, (**b**) the total number of rearing, and (**c**) the total number of grooming behaviors observed for 2 min for each observation day by four separation groups, the social housing (SH), no separation (NS), consecutive separation (CS), and intermittent separation (IS) (*^1^: NS > IS; Estimate ± SE = 0.65 ± 0.21, *z* = 3.18, *p* = 0.03, ***^2^: SH > CS; Estimate ± SE = 1.40 ± 0.32, *z* = 4.44, *p* < 0.001, ***^3^: NS > CS; Estimate ± SE = 1.97 ± 0.23, *z* = 8,44, *p* < 0.001, ***^4^: NS < IS; Estimate ± SE = 1.37 ± 0.21, *z* = 6.41, *p* < 0.001, **^5^: SH > CS; Estimate ± SE = 1.13 ± 0.31, *z* = 3.70, *p* = 0.004, **^6^: SH < IS; Estimate ± SE =  − 0.76 ± 0.24, *z* =  − 3.22, *p* = 0.02, *^7^: NS < CS; Estimate ± SE =  − 0.61 ± 0.20, *z* =  − 3.00, *p* = 0.049, ***^8^: NS < IS; Estimate ± SE =  − 0.85 ± 0.19, *z* =  − 4.38, *p* < 0.001, **^9^: NS < CS; Estimate ± SE =  − 0.62 ± 0.16, *z* =  − 3.80, *p* = 0.003, *^10^: NS < IS; Estimate ± SE =  − 0.51 ± 0.16, *z* = − 3.20, *p* = 0.02, *^11^: NS < CS; Estimate ± SE =  − 1.58 ± 0.48, *z* =  − 3.26, *p* = 0.02, multiple comparisons, *p < 0.05, **p < 0.01, and ***p < 0.001).

On PND 21, immediately after the end of the isolation experiment, behavioral differences were observed between the NS group and the CS and IS groups. The time spent freezing was longer in the NS group than in the IS group (Fig. 4a), whereas the total number of rearing behaviors was lower in the NS group than in the CS and IS groups (Fig. 4b). In addition, the total number of grooming behaviors was lower in the NS group than in the CS group (Fig. 4c). The total number of rearing behaviors was lower in the SH group than in the IS group (Fig. 4b).

Within a short time, the behavioral tendencies of each group started to vary. From PND21 to PND50, the time spent freezing increased in the NS group and largely decreased in the CS and IS groups than in the SH group (Fig. 4a, Supplementary Table S3; Separation group × PND). As a result, the time spent freezing tended to be longer in the SH and NS groups than in the CS and IS groups on PND 50 (Fig. 4a). In contrast, the total number of rearing behaviors increased in all groups, but was less in the IS groups than in the SH group during this period (Fig. 4b, Supplementary Table S4; Separation group × PND). Hence, the trend of the total number of rearing behaviors being lower in the NS group than in the CS and IS groups was maintained on PND50 (Fig. 4b).

**Figure 5:**
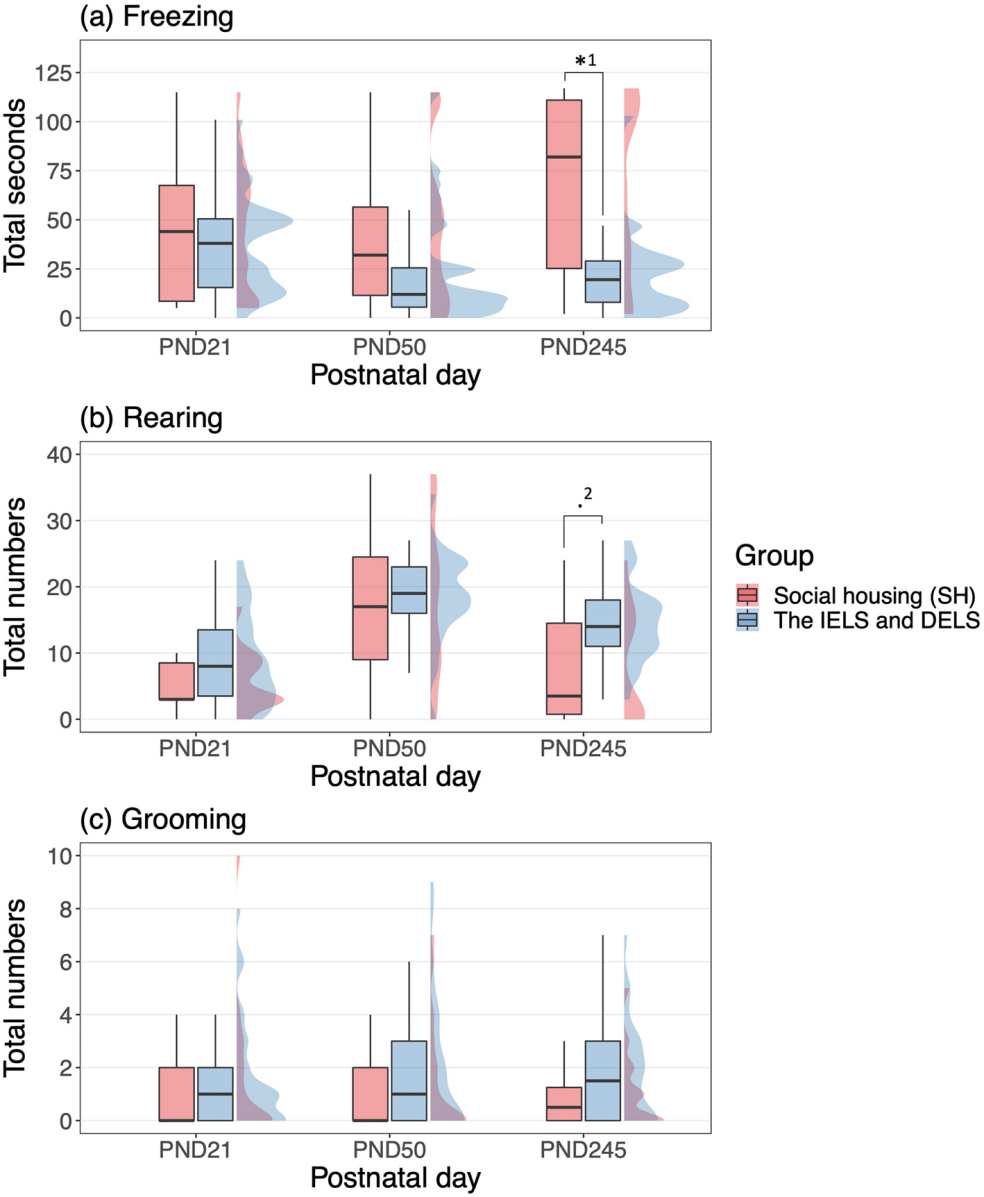
(**a**) Total time spent freezing, (**b**) the total number of rearing, and (**c**) the total number of grooming behaviors observed for 2 min for each observation day by the social housing (SH) and the IELS and DELS groups (no separation (NS), consecutive separation (CS), and intermittent separation (IS)) (*^1^: SH > NS, CS, IS; Estimate ± SE = 0.84 ± 0.29, *z* = 2.88, *p* = 0.01, .^2^: SH < NS, CS, IS; Estimate ± SE =  − 0.52 ± 0.23, *z* =  − 2.23, *p* = 0.08, multiple comparisons. *p* < 0.10 and *p < 0.05).

After a long time, the behavioral tendencies of each group were observed to have changed. From PND21 to PND245, the time spent freezing increased in the SH group than in the NS, CS and IS groups (Fig. 4a, Supplementary Table S3; Separation group × PND). Long time freezing was frequently observed only in the SH group. In contrast, the total number of rearing and grooming behaviors did not increase in the SH group compared to the NS group (Fig. 4b,c, Supplementary Tables S4, S5; Separation group × PND). Finally, the behavioral tendency of the NS group was closer to that of the CS and IS groups not to that of the SH group (Fig. 4). When, NS, CS, and IS groups were pooled into the IELS and DELS groups, the time spent freezing was longer in the SH group than in the IELS and DELS groups (Fig. 5a, Supplementary Table S6). In contrast, the total number of rearing behaviors tended to be lower in the SH group than in the IELS and DELS groups, although this was not statistically significant (Fig. 5b, Supplementary Table S6). In total, the IELS and DELS groups demonstrated hyperactivity.

### Sex differences between the effects of ELS and PND on behaviors

The time spent freezing was shorter in females than in males (Supplementary Table S7; Sex), whereas the total number of rearing and grooming behaviors was higher in females than in males (Supplementary Tables S8, S9; Sex). The difference in the time spent freezing between the CS and the SH groups was greater in males than in females (Supplementary Table S7; Separation group × Sex). The difference in the total number of rearing behaviors between the NS and the SH groups was greater in males than in females (Supplementary Table S8; Separation group × Sex). The difference in the total number of grooming behaviors between the IS and the SH groups was larger in males than in females (Supplementary Table S9; Separation group × Sex).

**Figure 6:**
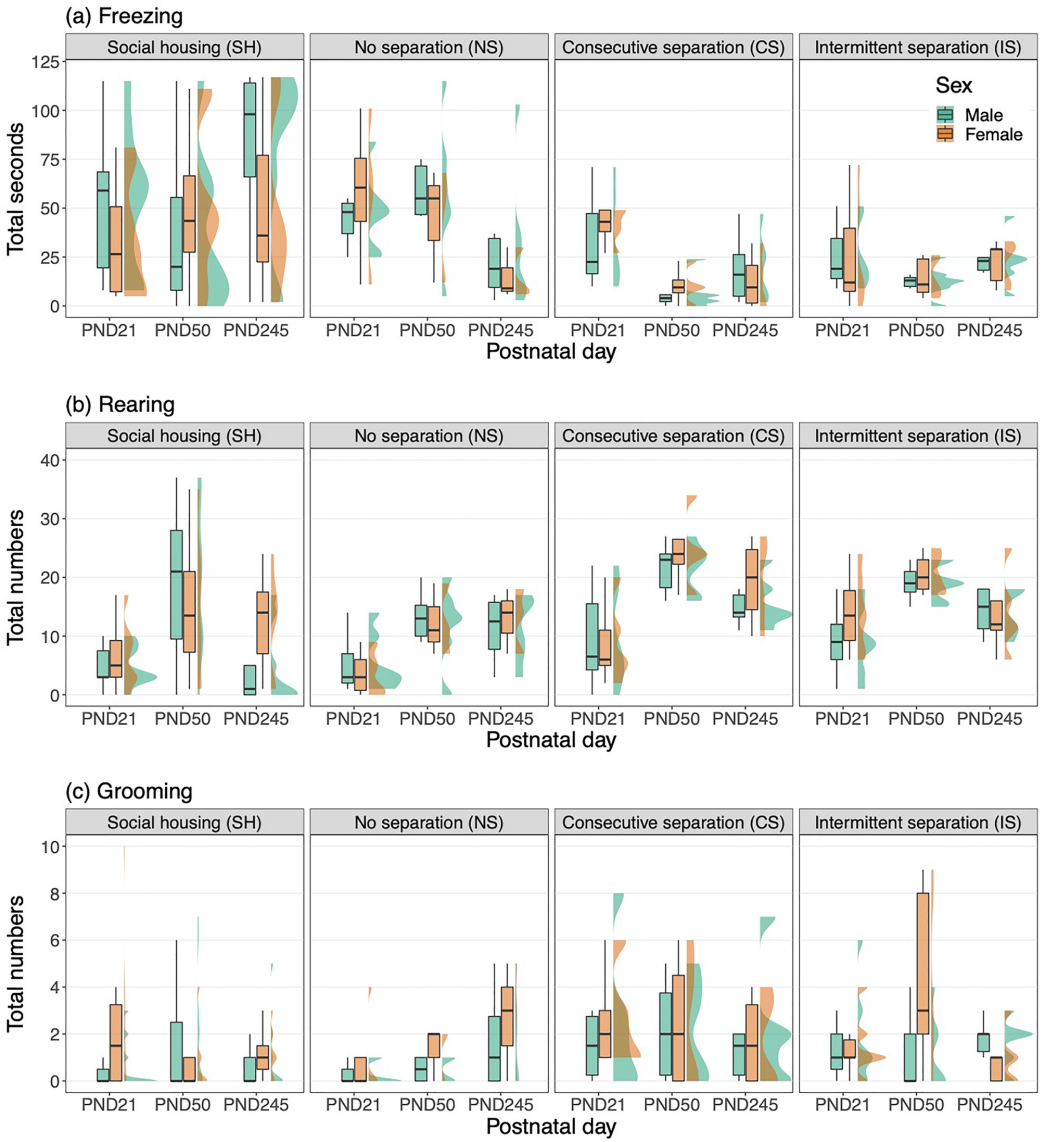
(**a**) Total time spent freezing, (**b**) the total number of rearing, and (**c**) the total number of grooming behaviors observed for 2 min for each observation day by sex and four separation groups, the social housing (SH), no separation (NS), consecutive separation (CS), and intermittent separation (IS).

Within a short time, from PND21 to PND50, the time spent freezing increased in females than in males in the SH group, while it decreased in females than in males in the NS and CS groups (Fig. 6, Supplementary Table S7; Separation group × PND × Sex). In contrast, the total number of rearing behaviors increased less in females than in males in the SH group, but did not increase in the NS and CS groups compared to the SH group (Fig. 6, Supplementary Table S8; Separation group × PND × Sex). The total number of grooming behaviors decreased in females than in males in the SH group, but did not decrease in the CS groups, and increased in females compared to males in the IS group (Fig. 6, Supplementary Table S9; Separation group × PND × Sex).

After a long time, from PND21 to PND245, the time spent freezing increased in males than in females in the SH group, while it decreased in females than in males in the NS and CS groups (Fig. 6, Supplementary Table S7; Separation group × PND × Sex). The total number of rearing behaviors increased in females than in males in the SH group while it did not increase in the IS group (Fig. 6, Supplementary Table S8; Separation group × PND × Sex). The time spent freezing increased in males than in females in the SH group (Supplementary Table S10a; Separation group × PND × Sex), while it decreased in both sexes in the IELS and DELS groups (Fig. 6a). In contrast, the total number of rearing behaviors decreased in males than in females in the SH group (Supplementary Table S10b; Separation group × PND × Sex), while both sexes increased or demonstrated similar levels in the IELS and DELS groups (Fig. 6b). Overall, sex differences in the time spent freezing and the total number of rearing behaviors were very small throughout the study period in the IELS and DELS groups (Fig. 6a,b).

## Discussion

### Effects of ELS on bodyweight gain

Our results indicate that ELS does not affect bodyweight gain. Previous studies conducted on *O*. *degus* reported that 1-h of daily parental separation immediately affected the plasma levels of glucocorticoids, a key indicator of stress, during the first 3 weeks of life^33^, whereas the effects of stress do not manifest in the bodyweight^39^. Uekita^40^ also reported that ELS did not affect the bodyweight of degus for 6 weeks post-birth, and there were no significant effects of ELS on mother-infant interactions, which indicate that no malnutrition occurred, nor did the infant experience neglect by the mother. Similar to these previous studies^39^, Uekita^40^, our results suggest that ELS does not cause malnutrition in degus or neglect by their mothers during the early development period. In contrast, the mean body weight of males and females in the SH group was higher than that in the other groups from PND35 to PND245. Therefore, there is the possibility that ELS will affect bodyweight gain after weaning. As the bodyweight of degus continues to increase in captivity beyond the experimental period, it will be necessary to continue observing the animals until the weight gain reaches a plateau. This will help determine whether these observed differences will affect the degree of development or whether they will slow down and eventually disappear with time.

### Short-term effects of ELS and PND on behaviors

During a short period of observation, the time spent freezing tended to be longer in the SH and NS groups than in the CS and IS groups, while the total number of rearing behaviors was lower in the NS group than in the CS and IS groups. These results indicate that the CS and IS groups that experienced DELS were more active. This shows the same trend as the previous reports^33,37,39–42^. In addition, Degu is the most active on PND50. There are no comprehensive reports describing the activity according to the developmental stage in degus, but in mice, the behavior changes are age-related^43,44^. Since sexual maturity in degus is observed as early as about 2 months of age^45^, PND50 is a pre-sexual stage, and the trend of declining activity after sexual maturity is consistent with findings in mice. Nevertheless, the DELS group was more active than the SH and NS groups on PND 50. Therefore, basically, our results efficiently demonstrated the effects of ELS on the behaviors of separated animals reported in previous studies and confirmed that the DELS group displayed hyperactivity. In contrast, our results also suggest that the behavioral tendencies were similar between the SH group and the NS (IELS) groups within a short observation period.

### Long-term effects of ELS and PND on behaviors

In the animal life cycle, the period after sexual maturation is typically longer than the early development period, and there are many events that occur to ensure preservation of the next generation. Whether the effects of ELS manifest after sexual maturation or not should be examined. However, reports on the long-term effects of ELS are lacking, thus, our study has a pioneering effect in shedding light on these effects. During a long observation period, the DELS group was more active than the SH group. Hence, the behavioral effects of ELS can be interpreted as long-term preservation. Rivera^38^ reported on the long-lasting effects of ELS and showed that there were no differences among the groups at the age of 31 months, which is contrary to the results of the present study. This could be due to differences in observation parameters and the age of the animals. Alternatively, they reported that learning ability and memory were the lowest in the group that had been kept alone after separation, and these performances fully recovered at the age of 31 months when the rearing method of the single-reared animals was changed to group rearing after the behavioral test conducted at the age of 25 months. The long-term effects on learning ability and memory resemble the long-term effects on the behavioral parameters in this study.

Surprisingly, the time spent freezing decreased and the total number of rearing behaviors increased in the NS group compared to the SH group, resulting in the behavioral tendencies of the NS group that did not experience direct separation, being closer to those of the two hyperactive groups that did experience separation. Compared to the SH group, the NS, CS, and IS groups have relatively small sample sizes. However, this trend remains the same when comparing the SH group and the IELS and DELS group (Fig. 5). Therefore, we hypothesized that the NS (IELS) group may be indirectly affected by the ELS. We propose three possible stressors. The first is communication with the DELS animals. Since degus are social animals, interaction with other individuals is an important factor^35^. We did not separate pups chronically in this study, so the degus had social interactions with their siblings outside the social separation period. The difference between the SH and NS (IELS) groups is related to whether the cage mate siblings have experienced parental separation or not. Endo^45^ reported that when group-housed and socially isolated adult mice were co-housed, the isolated mice group was slowest to exhibit huddling behavior with other individuals. Moreover, they reported that the number of times that isolated mice approached group-housed mice was greater than the reverse approach behavior. This suggests that separated individuals may become over-communicative. To confirm that communications with the DELS animals will be a stressor for the NS (IELS) group, we should identify whether individuals who have experienced DELS could establish appropriate relationships with cohabitant relatives or not. The second is the experience of temporarily losing siblings, and the third is having heard the DVs of the separated siblings. Degus live in groups and communicate through vocalization^25,26^. Thus, the NS (IELS) group might have been stressed by the group that experienced separation and by the DVs emitted by these animals during the period when only the CS group was separated (Fig. 2; PND4–7, 9–13, 15–20). If the NS (IELS) animals felt stressed by these, the NS (IELS) group may feel empathy for the stress of the DELS animals, and the IS group could have been similarly stressed when only the CS group was separated. In this study, the behavioral tendency of the NS (IELS) group resembled that of the SH group on PND21, but it became closer to those of the DELS group on PND245 (Fig. 6). Therefore, we need to investigate whether the second and third stressors may act in a delayed manner.

### Sex differences in the effects of ELS and PND on behaviors

In this study, females were more active than males, whereas the differences between the groups were larger in males than in females. This suggests that the effects of ELS were greater in males than in females. Gruss^33^ also reported that while females were more active, including in the control group, males showed greater group differences and were more hyperactive in the group that experienced separation, supporting the trend in this study. Additionally, ELS affects the development of catecholaminergic systems, and ELS-induced differences in tyrosine hydroxylase-immunoreactive fibers were more pronounced in male brains than in female brains^47^. Furthermore, we observed that the difference in behavioral tendencies between males and females was larger in the SH group than in the NS, CS, and IS groups, suggesting that DELS and IELS may converge an individual’s behavioral tendencies in a certain direction.

### Summary

**Figure 7:**
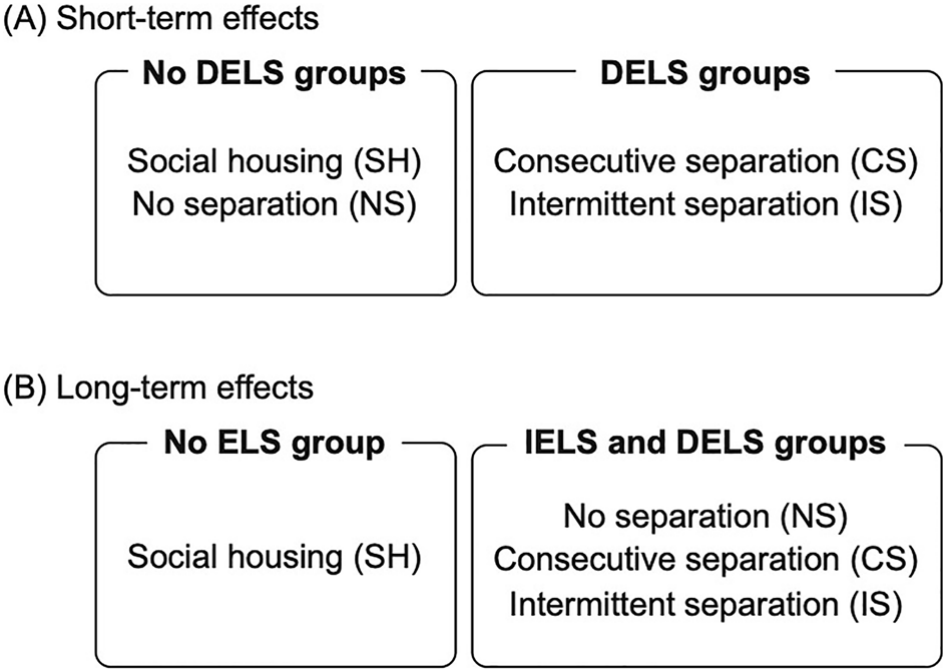
Diagram of grouping by trends in the results found in this study. The trends of short-term effects were divided by the degus experienced DELS or not (**A**), but the trends of long-term effects indicated that individuals in the NS (IELS) group exhibited changes in behavioral trends similar to those who experienced separation (DELS) later in life (**B**).

We illustrated that ELS has both "direct" and "indirect" effects on degus, resulting in changes in their behavioral trends and increasing their susceptibility to stressors. DELS was correlated with higher hyperactivity through the experimental period, and the hyperactivity increased with more frequent separation. A noteworthy finding is that individuals in the NS (IELS) group, who did not experience parental separation themselves, but had siblings who did, exhibited changes in behavioral trends similar to those who experienced separation (DELS) later in life (Fig. 7). In addition, we observed differences in the pattern of changes in behavioral tendencies between males and females across groups, with the largest differences between the sexes observed in the SH group compared to the DELS and IELS groups. Future studies focusing on the effects of IELS as well as DELS with respect to sex will provide insights into the underlying stressors or factors behind the development of anxiety or stress-related behaviors in social animals, which in turn will contribute to the development of animal welfare measures of domestic animals, laboratory animals, zoo animals, and livestock.

## Methods

### Animals and housing conditions

The degus were bred as a colony at the Miyazaki University Environmental Control Laboratory, Faculty of Agriculture, sources from a colony at the Miyazaki University Frontier Science Research Center. The colonies were maintained by pairing offspring from different parents to obtain litters. The groups used in the present experiment were produced in the same way, except for one of the SH groups (a control group) that had pups born from the same littermate for the purpose of another experiment, and these were included in the observations. The influence of the family group was examined by statistical analysis. The degus were bred at the Environmental Control room 1 (room 1), Faculty of Agriculture, University of Miyazaki. Room 1 was maintained at 22 ± 2 °C, with a 14:10 h light/dark cycle from January 26, 2017, to May 26, 2019, and a 12:12 h light/dark cycle from May 27, 2019, to November 07, 2019, with the light on starting at 08:00 JST (Japan Standard Time: UTC + 9). A female and a male were paired in a plastic cage (34.5 cm l × 40.3 cm w × 17.7 cm h) with wood chips for bedding, which is defined as a home cage. Commercial rodent diets (Labo MR Stock and Labo G Standard, Nosan Corporation, Kanagawa, Japan) and water were provided ad libitum. Each breeding couple was checked for a new litter daily, and the male was removed from the home cage after the presence of a litter was confirmed. Litter size was not standardized (three to seven pups per litter), and the degus were maintained as a family group composed of one mother and her pups before weaning on PND35. Their siblings were reared in the same cage until PND50, after which up to three siblings of the same sex were reared in the same cage. For example, the litter member was composed of three individuals: two males and a female divided between two male cages and only one female cage. In another pattern, the litter consisted of seven individuals, comprising two males and five females, divided into two male cages and two, and three female cages. In the SH group, the animals were kept with other SH groups of the same sex. In the other three groups, no more than two individuals from the same group were included in the same cage. Four experimental groups (Fig. 2) were considered in this study: (i) the SH group (a control group); a whole litter that did not experience any separation treatment. Conversely, the litters were randomly assigned to three groups formed among the siblings to avoid the influence of bias in temperament and characteristics of each dam among the families and according to the following treatments (for the number of animals and litters used at each age, see Supplementary Table S1): (ii) NS group; the pups did not experience separation from their mother, but their siblings did, (iii) CS group; the pups were removed from their mother and home cage for 1-h every day, (iv) IS group; the pups were removed from their mother and home cage for 1-h on PND3, PND8, and PND14. To minimize the stresses from direct handling, the pups were identified by ear punching when they experienced the first handling treatment; the animals in the CS and IS groups were ear-punched after the separation on PND3, and the animals in the SH and NS groups were ear-punched after the OFT on PND21.

All procedures adhered to national and institutional guidelines and were approved by the Institutional Animal Experimentation Committee at the University of Miyazaki (Permission Nos. 2017-025-1 to 3 and 2020-012-1) and were performed in accordance with the ARRIVE guidelines^48^.

### Separation treatments and open-field tests (Fig. 2)

The separation was defined as when a subject was housed alone for 1-h in a novel white opaque plastic rodent cage (34.5 cm l × 40.3 cm w × 17.7 cm h). On PND3, PND8, and PND14, the CS and IS pups were separated and placed in the Environmental Control room 2 (room 2) maintained at 22 ± 2 °C, and under light conditions. Thus, during the separation, the separated pups could communicate with separated siblings using acoustic and olfactory cues; however, no visual or social contact (touching) could be established with separated siblings in room 2, or with their siblings and parents in their home cages in room 1. On PND4–7, PND9–13, and PND15–20, the CS group pups were kept individually in a novel white opaque plastic cage for 1-h daily in room 1. Thus, during the separation, the pups had acoustic and olfactory, but no visual or social contact with their siblings and parents. After 24-h separation, the pups were returned to their home cages and left undisturbed until the next separation. All groups were tested using the OFT in a novel cage (34.5 cm l × 40.3 cm w × 60.0 cm h) in room 2 for 1-h on PND21 and PND50, and for 20 min on PND245. The behaviors of the animals were recorded with a digital video camera (Panasonic HX-A1H). All experimental procedures were carried out between 10:00 and 16:00 during the peak activity phase of this day-active species. All subjects were selected randomly at different times to control for any possible sequence effects of sex and treatment.

### Bodyweight measuring

Bodyweights, recorded to the nearest 0.1 g, were used as a general indicator of stress responsivity after the animals of CS and IS were separated on PND3, PND8, and PND14. All animals were separated and weighed on PND21, PND50, and PND245 for behavioral observations. NS was performed on PND35 and PND64, but their weights were measured.

### Observation of behavior (PND21, PND50, and PND245)

In OFT, to observe the stress responses of animals when introduced into a novel environment, behavioral analysis was conducted during the first 2 min after the animal was introduced into a novel cage. The recorded videos of the animal behaviors were visually examined, and using the previously described method^49^, the following three parameters that may indicate a stress response were recorded: (1) freezing: time the animals spent being immobile ≥ 2 s; (2) rearing: number of instances where the animals stood on their hind legs, with or without contacting the sides of the box; and (3) grooming: number of self-grooming behaviors.

### Statistical analyses

All statistical analyses were performed using R version 4.1.0 (Core Team, 2021). Factors affecting freezing behavior and rearing behavior and bodyweight were analyzed with a generalized linear mixed model (GLMM) using the GLMM function of the lme4 package 1.1-27-1^50^. In the analysis of bodyweight, separation treatments (SH, NS, CS, and IS), PND (from PND21–PND245), and sex and the interactions between the separation treatments and PND, separation treatments and sex, PND and sex, separation treatments, and PND and sex were treated as fixed effects. First, in the analysis of behavior, we analyzed the separation treatments (SH, NS, CS, and IS) and PND (PND21, PND50, and PND245), and the interactions between the separation treatments and PND were treated as fixed effects to compare the trends of each PND. The results of the analysis of the models were analyzed using the multiple comparison method with the least squares averaging to compare the values between the separated conditions at each age^51^. All pairwise comparisons were Bonferroni corrected. In addition, the NS, CS, and IS groups were pooled into the IELS and DELS groups and compared to the SH group using the same procedure. Second, we analyzed the separation treatments (SH, NS, CS, and IS) and PND (PND21, PND50, and PND245), and the interactions between the separation treatments and PND, separation treatments and sex, PND and sex, separation treatments, and PND and sex were treated as fixed effects to consider the sex effects. The NS, CS, and IS groups were pooled into the IELS and DELS groups and compared to the SH group using the same procedure. The statistical analyses accounted for repeated observations of the same individuals and potential family-level effects by including “individual” and “family” as random effects. In advance, the effects of the number of litter mates and the number of cohabitants after weaning were analyzed and excluded because those did not affect the trend of results. We selected the simplest models with the lowest Akaike’s information criterion. For all statistical tests, the level of significance was set at *p* < 0.05.

## Supplementary Information

Supplementary Tables.

## Data Availability

The datasets generated during and/or analyzed during the current study are available from the corresponding author on reasonable request.
