# Supplementary material for: Long-term behavioral effects of social separation during early life in a social mammal, Octodon degus
Source: Sci Rep. 2023 Jun 12;13:9518. doi: 10.1038/s41598-023-36745-6 (PMC10261089; doi:10.1038/s41598-023-36745-6)
Supplement: Supplementary file 1 — Supplementary Tables. [file 41598_2023_36745_MOESM1_ESM.docx]

**Long-term behavioral effects of social separation during early life in a social mammal, *Octodon degus***

**Authors**

Rina Ukyo^1^, Akio Shinohara^2^, Chihiro Koshimoto^2^, Goro A. Nagura-Kato^2^, Seiji Ieiri^3^, Yasuhiro Tsuzuki^3^, Shinsuke H. Sakamoto^3,^ *

**Affiliations**

^1^ Interdisciplinary Graduate School of Agriculture and Engineering, University of Miyazaki

^2^ Division of Bio-Resources, Department of Biotechnology, Frontier Science Research Center, University of Miyazaki, 5200 Kihara, Kiyotake-cho, Miyazaki-shi, Miyazaki, 889-1692, Japan

^3^ Faculty of Agriculture, University of Miyazaki, Miyazaki 889-2192, Japan

**Supplementary Information**

**Table S1.** Number of individuals (males/females) and litters (given in parentheses) in each group.

|  | Age |  | Group | | | |  |
| --- | --- | --- | --- | --- | --- | --- | --- |
|  |  |  | Social housing (SH) | No separation (NS) | Consecutive separation (CS) | Intermittent separation (IS) |  |
|  |  |  |  |  |  |  |  |
|  |  |  |  |  |  |  |  |
|  | PND3 |  | 11/8 (6) | 7/4 (7) | 6/5 (7) | 7/6 (7) |  |
|  | PND21 |  | 11/8 (6) | 7/4 (7) | 6/5 (7) | 7/6 (7) |  |
|  | PND35 |  | 11/8 (6) | 7/4 (7) | 6/5 (7) | 7/6 (7) |  |
|  | PND50 |  | 11/8 (6) | 6/3 (6) | 6/4 (6) | 7/5 (6) |  |
|  | PND64 |  | 11/8 (6) | 6/3 (6) | 6/4 (6) | 6/5 (6) |  |
|  | PND245 | | 10/8 (6) | 6/3 (6) | 6/4 (6) | 6/5 (6) |  |

**Table S2.** The effects of ELS and PND on the bodyweight gaining demonstrated by four separation groups, the social housing (SH), no separation (NS), consecutive separation (CS), and intermittent separation (IS).

| Variables |  |  |  | Estimate | ± | SE | *t* | Pr(>\|*t*\|) |
| --- | --- | --- | --- | --- | --- | --- | --- | --- |
| Separation group | | |  |  |  |  |  |  |
| NS |  |  |  | −4.11 | ± | 6.15 | −0.67 | 0.51 |
| CS |  |  |  | −6.34 | ± | 6.21 | −1.02 | 0.32 |
| IS |  |  |  | −7.68 | ± | 6.17 | −1.24 | 0.23 |
| PND |  |  |  |  |  |  |  |  |
| PND | | |  | 0.61 | ± | 0.01 | 41.29 | **<0.001** |
| Sex |  |  |  |  |  |  |  |  |
| Female | | |  | 3.42 | ± | 3.92 | 0.87 | 0.38 |
|  |  |  |  |  |  |  |  |  |
| Separation group × Sex | | | |  |  |  |  |  |
| NS | × | Female |  | −4.62 | ± | 5.72 | −0.81 | 0.42 |
| CS | × | Female |  | −0.92 | ± | 5.65 | −0.16 | 0.87 |
| IS | × | Female |  | 2.36 | ± | 5.34 | 0.44 | 0.66 |
|  |  |  |  |  |  |  |  |  |
| PND × Sex | | | |  |  |  |  |  |
| PND | × | Female |  | −0.08 | ± | 0.02 | −3.57 | **<0.001** |

**Table S3.** The effects of ELS and PND on the time spent freezing behaviors demonstrated by four separation groups, the social housing (SH), no separation (NS), consecutive separation (CS), and intermittent separation (IS).

| Variables |  |  |  | Estimate | ± | SE | *z* | Pr(>\|*z*\|) |
| --- | --- | --- | --- | --- | --- | --- | --- | --- |
| Separation group | | |  |  |  |  |  |  |
| NS |  |  |  | 0.26 | ± | 0.30 | 0.87 | 0.39 |
| CS |  |  |  | −0.02 | ± | 0.30 | −0.07 | 0.94 |
| IS |  |  |  | −0.39 | ± | 0.30 | −1.33 | 0.18 |
| PND |  |  |  |  |  |  |  |  |
| 50 | | |  | −0.07 | ± | 0.05 | −1.47 | 0.14 |
| 245 | | |  | 0.34 | ± | 0.05 | 7.28 | **<0.001** |
|  |  |  |  |  |  |  |  |  |
| Separation group × PND | | | |  |  |  |  |  |
| NS | × | 50 |  | 0.31 | ± | 0.08 | 3.63 | **<0.001** |
| CS | × | 50 |  | −1.38 | ± | 0.13 | −10.50 | **<0.001** |
| IS | × | 50 |  | −0.41 | ± | 0.11 | −3.65 | **<0.001** |
| NS | × | 245 |  | −0.83 | ± | 0.09 | −8.81 | **<0.001** |
| CS | × | 245 |  | −1.11 | ± | 0.11 | −10.55 | **<0.001** |
| IS | × | 245 |  | −0.30 | ± | 0.10 | −3.01 | **0.003** |

**Table S4.** The effects of ELS and PND on the total number of rearing behaviors demonstrated by four separation groups, the social housing (SH), no separation (NS), consecutive separation (CS), and intermittent separation (IS).

| Variables |  |  |  | Estimate | ± | SE | *z* | Pr(>\|*z*\|) |
| --- | --- | --- | --- | --- | --- | --- | --- | --- |
| Separation group | | |  |  |  |  |  |  |
| NS |  |  |  | −0.08 | ± | 0.26 | −0.31 | 0.76 |
| CS |  |  |  | 0.53 | ± | 0.25 | 2.14 | **0.03** |
| IS |  |  |  | 0.76 | ± | 0.24 | 3.22 | **0.001** |
| PND |  |  |  |  |  |  |  |  |
| 50 | | |  | 1.15 | ± | 0.11 | 10.29 | **<0.001** |
| 245 | | |  | 0.41 | ± | 0.13 | 3.12 | **0.002** |
|  |  |  |  |  |  |  |  |  |
| Separation group × PND | | | |  |  |  |  |  |
| NS | × | 50 |  | −0.30 | ± | 0.20 | −1.47 | 0.14 |
| CS | × | 50 |  | −0.30 | ± | 0.16 | −1.76 | 0.08 |
| IS | × | 50 |  | −0.63 | ± | 0.15 | −4.13 | **<0.001** |
| NS | × | 245 |  | 0.43 | ± | 0.22 | 1.97 | **0.048** |
| CS | × | 245 |  | 0.15 | ± | 0.18 | 0.82 | 0.41 |
| IS | × | 245 |  | −0.23 | ± | 0.18 | −1.32 | 0.19 |

**Table S5.** The effects of ELS and PND on the total number of grooming behaviors demonstrated by four separation groups, the social housing (SH), no separation (NS), consecutive separation (CS), and intermittent separation (IS).

| Variables |  |  |  | Estimate | ± | SE | *z* | Pr(>\|*z*\|) |
| --- | --- | --- | --- | --- | --- | --- | --- | --- |
| Separation group | | |  |  |  |  |  |  |
| NS |  |  |  | −0.81 | ± | 0.61 | −1.32 | 0.19 |
| CS |  |  |  | 0.77 | ± | 0.50 | 1.54 | 0.12 |
| IS |  |  |  | 0.33 | ± | 0.51 | 0.65 | 0.52 |
| PND |  |  |  |  |  |  |  |  |
| 50 | | |  | −4.67 | ± | 0.27 | −0.00 | 1.00 |
| 245 | | |  | −0.11 | ± | 0.32 | −0.35 | 0.72 |
|  |  |  |  |  |  |  |  |  |
| Separation group × PND | | | |  |  |  |  |  |
| NS | × | 50 |  | 0.26 | ± | 0.61 | 0.43 | 0.67 |
| CS | × | 50 |  | −0.11 | ± | 0.39 | −0.28 | 0.78 |
| IS | × | 50 |  | 0.40 | ± | 0.39 | 1.04 | 0.30 |
| NS | × | 245 |  | 1.32 | ± | 0.56 | 2.34 | **0.02** |
| CS | × | 245 |  | −0.19 | ± | 0.43 | −0.43 | 0.67 |
| IS | × | 245 |  | −0.12 | ± | 0.46 | −0.26 | 0.79 |

**Table S6.** The effects of ELS and PND on (a) the total time spent freezing, (b) the total number of rearing, and (c) the total number of grooming behaviors demonstrated by the social housing (SH) and the IELS and DELS groups (no separation (NS), consecutive separation (CS), and intermittent separation (IS)).

| Models | Variables | |  |  | Estimate | ± | SE | *z* | Pr(>\|*z*\|) |
| --- | --- | --- | --- | --- | --- | --- | --- | --- | --- |
| (a) Freezing behavior | | |  |  |  |  |  |  |  |
|  | Separation group | | |  |  |  |  |  |  |
|  |  | NS, CS, IS |  |  | −0.09 | ± | 0.29 | −0.3 | 0.76 |
|  | PND | |  |  |  |  |  |  |  |
|  |  | 50 | |  | −0.07 | ± | 0.05 | −1.48 | 0.14 |
|  |  | 245 | |  | 0.34 | ± | 0.05 | 7.26 | **<0.001** |
|  |  |  |  |  |  |  |  |  |  |
|  | Separation group × PND | | | |  |  |  |  |  |
|  |  | NS, CS, IS | × | 50 | −0.24 | ± | 0.07 | −3.45 | **<0.001** |
|  |  | NS, CS, IS | × | 245 | −0.75 | ± | 0.07 | −10.94 | **<0.001** |
|  |  |  |  |  |  |  |  |  |  |
| (b) Rearing behavior | | |  |  |  |  |  |  |  |
|  | Separation group | | |  |  |  |  |  |  |
|  |  | NS, CS, IS |  |  | 0.48 | ± | 0.24 | 2.02 | **0.04** |
|  | PND | |  |  |  |  |  |  |  |
|  |  | 50 | |  | 1.15 | ± | 0.11 | 10.3 | **<0.001** |
|  |  | 245 | |  | 0.42 | ± | 0.13 | 3.12 | **0.002** |
|  |  |  |  |  |  |  |  |  |  |
|  | Separation group × PND | | | |  |  |  |  |  |
|  |  | NS, CS, IS | × | 50 | −0.45 | ± | 0.13 | −3.36 | **<0.001** |
|  |  | NS, CS, IS | × | 245 | 0.04 | ± | 0.15 | 0.27 | 0.79 |
|  |  |  |  |  |  |  |  |  |  |
| (c) Grooming behavior | | |  |  |  |  |  |  |  |
|  | Separation group | | |  |  |  |  |  |  |
|  |  | NS, CS, IS |  |  | 0.29 | ± | 0.46 | 0.62 | 0.53 |
|  | PND | |  |  |  |  |  |  |  |
|  |  | 50 | |  | 0.00 | ± | 0.27 | 0.00 | 1.00 |
|  |  | 245 | |  | −0.10 | ± | 0.32 | −0.3 | 0.76 |
|  |  |  |  |  |  |  |  |  |  |
|  | Separation group × PND | | | |  |  |  |  |  |
|  |  | NS, CS, IS | × | 50 | 0.17 | ± | 0.33 | 0.51 | 0.61 |
|  |  | NS, CS, IS | × | 245 | 0.14 | ± | 0.37 | 0.38 | 0.70 |

**Table S7.** The effects of ELS, PND, and sex on the time spent freezing behaviors demonstrated by four groups, the social housing (SH), no separation (NS), consecutive separation (CS), and intermittent separation (IS).

| Variables |  |  |  |  | Estimate | ± | SE | *z* | Pr(>\|*z*\|) |
| --- | --- | --- | --- | --- | --- | --- | --- | --- | --- |
| Separation group | | |  |  |  |  |  |  |  |
| NS |  |  |  |  | −0.03 | ± | 0.34 | −0.07 | 0.94 |
| CS |  |  |  |  | −0.42 | ± | 0.35 | −1.20 | 0.23 |
| IS |  |  |  |  | −0.46 | ± | 0.34 | −1.32 | 0.19 |
| PND |  |  |  |  |  |  |  |  |  |
| 50 | | |  |  | −0.43 | ± | 0.07 | −6.38 | **<0.001** |
| 245 | | |  |  | 0.33 | ± | 0.06 | 5.75 | **<0.001** |
| Sex |  |  |  |  |  |  |  |  |  |
| Female | | |  |  | −0.57 | ± | 0.25 | −2.27 | **0.02** |
|  |  |  |  |  |  |  |  |  |  |
| Separation group × PND | | | |  |  |  |  |  |  |
| NS | × | 50 |  |  | 0.76 | ± | 0.11 | 7.13 | **<0.001** |
| CS | × | 50 |  |  | −1.16 | ± | 0.19 | −6.28 | **<0.001** |
| IS | × | 50 |  |  | −0.28 | ± | 0.15 | −1.90 | 0.06 |
| NS | × | 245 |  |  | −0.61 | ± | 0.11 | −5.50 | **<0.001** |
| CS | × | 245 |  |  | −0.89 | ± | 0.13 | −6.77 | **<0.001** |
| IS | × | 245 |  |  | −0.46 | ± | 0.13 | −3.59 | **<0.001** |
|  |  |  |  |  |  |  |  |  |  |
| Separation group × Sex | | | |  |  |  |  |  |  |
| NS | × | Female |  |  | 0.75 | ± | 0.40 | 1.89 | 0.06 |
| CS | × | Female |  |  | 0.95 | ± | 0.40 | 2.36 | **0.02** |
| IS | × | Female |  |  | 0.20 | ± | 0.39 | 0.50 | 0.62 |
|  |  |  |  |  |  |  |  |  |  |
| PND × Sex | |  |  |  |  |  |  |  |  |
| 50 | × | Female |  |  | 0.84 | ± | 0.10 | 8.14 | **<0.001** |
| 245 | × | Female |  |  | 0.04 | ± | 0.10 | 0.41 | 0.68 |
|  |  |  |  |  |  |  |  |  |  |
| Separation group × PND × Sex | | | | |  |  |  |  |  |
| NS | × | 50 | × | Female | −1.15 | ± | 0.18 | −6.43 | **<0.001** |
| CS | × | 50 | × | Female | −0.56 | ± | 0.27 | −2.10 | **0.04** |
| IS | × | 50 | × | Female | −0.27 | ± | 0.23 | −1.18 | 0.24 |
| NS | × | 245 | × | Female | −0.84 | ± | 0.22 | −3.80 | **<0.001** |
| CS | × | 245 | × | Female | −0.59 | ± | 0.22 | −2.66 | **0.008** |
| IS | × | 245 | × | Female | 0.40 | ± | 0.21 | 1.90 | 0.06 |

**Table S8.** The effects of ELS, PND, and sex on the number of rearing behaviors demonstrated by four groups, the social housing (SH), no separation (NS), consecutive separation (CS), and intermittent separation (IS).

| Variables |  |  |  |  | Estimate | ± | SE | *z* | Pr(>\|*z*\|) |
| --- | --- | --- | --- | --- | --- | --- | --- | --- | --- |
| Separation group | | |  |  |  |  |  |  |  |
| NS |  |  |  |  | 0.25 | ± | 0.31 | 0.79 | 0.43 |
| CS |  |  |  |  | 0.76 | ± | 0.30 | 2.56 | **0.01** |
| IS |  |  |  |  | 0.72 | ± | 0.29 | 2.48 | **0.01** |
| PND |  |  |  |  |  |  |  |  |  |
| 50 |  |  |  |  | 1.40 | ± | 0.15 | 9.09 | **<0.001** |
| 245 |  |  |  |  | 0.01 | ± | 0.21 | 0.05 | 0.96 |
| Sex |  |  |  |  |  |  |  |  |  |
| Female | | |  |  |  |  | 0.23 | 2.00 | **0.046** |
|  |  |  |  |  |  |  |  |  |  |
| Separation group × PND | | | | |  |  |  |  |  |
| NS | × | 50 |  |  | −0.64 | ± | 0.26 | −2.51 | **0.01** |
| CS | × | 50 |  |  | −0.58 | ± | 0.22 | −2.61 | **<0.001** |
| IS | × | 50 |  |  | −0.66 | ± | 0.22 | −3.06 | **0.002** |
| NS | × | 245 |  |  | 0.70 | ± | 0.30 | 2.37 | **0.02** |
| CS | × | 245 |  |  | 0.48 | ± | 0.27 | 1.77 | 0.08 |
| IS | × | 245 |  |  | 0.44 | ± | 0.27 | 1.63 | 0.10 |
|  |  |  |  |  |  |  |  |  |  |
| Separation group × Sex | | | | |  |  |  |  |  |
| NS | × | Female |  |  | −0.83 | ± | 0.41 | −2.02 | **0.04** |
| CS | × | Female |  |  | −0.54 | ± | 0.34 | −1.57 | 0.12 |
| IS | × | Female |  |  | 0.00 | ± | 0.32 | 0.01 | 1.00 |
|  |  |  |  |  |  |  |  |  |  |
| PND × Sex | | | | |  |  |  |  |  |
| 50 | × | Female |  |  | −0.58 | ± | 0.23 | −2.57 | **0.01** |
| 245 | × | Female |  |  | 0.61 | ± | 0.28 | 2.20 | **0.03** |
|  |  |  |  |  |  |  |  |  |  |
| Separation group × PND × Sex | | | | |  |  |  |  |  |
| NS | × | 50 | × | Female | 0.86 | ± | 0.43 | 1.99 | **0.047** |
| CS | × | 50 | × | Female | 0.67 | ± | 0.33 | 2.01 | **0.04** |
| IS | × | 50 | × | Female | 0.14 | ± | 0.31 | 0.45 | 0.65 |
| NS | × | 245 | × | Female | −0.23 | ± | 0.46 | −0.51 | 0.61 |
| CS | × | 245 | × | Female | −0.44 | ± | 0.37 | −1.17 | 0.24 |
| IS | × | 245 | × | Female | −1.15 | ± | 0.36 | −3.18 | **0.001** |

**Table S9.** The effects of ELS, PND, and sex on the number of grooming behaviors demonstrated by four groups, the social housing (SH), no separation (NS), consecutive separation (CS), and intermittent separation (IS).

| Variables |  |  |  |  | Estimate | ± | SE | *z* | Pr(>\|*z*\|) |
| --- | --- | --- | --- | --- | --- | --- | --- | --- | --- |
| Separation group | | |  |  |  |  |  |  |  |
| NS |  |  |  |  | −0.55 | ± | 0.91 | −0.61 | 0.54 |
| CS |  |  |  |  | 1.57 | ± | 0.65 | 2.41 | **0.02** |
| IS |  |  |  |  | 1.25 | ± | 0.66 | 1.90 | **0.06** |
| PND |  |  |  |  |  |  |  |  |  |
| 50 |  |  |  |  | 1.20 | ± | 0.45 | 2.65 | **0.008** |
| 245 |  |  |  |  | 0.74 | ± | 0.54 | 1.38 | 0.17 |
| Sex |  |  |  |  |  |  |  |  |  |
| Female | | |  |  | 1.41 |  | 0.53 | 2.64 | **0.008** |
|  |  |  |  |  |  |  |  |  |  |
| Separation group × PND | | | | |  |  |  |  |  |
| NS | × | 50 |  |  | −0.71 | ± | 1.00 | −0.71 | 0.48 |
| CS | × | 50 |  |  | −1.28 | ± | 0.59 | −2.17 | **0.03** |
| IS | × | 50 |  |  | −1.61 | ± | 0.64 | −2.53 | **0.01** |
| NS | × | 245 |  |  | 0.95 | ± | 0.93 | 1.03 | 0.30 |
| CS | × | 245 |  |  | −0.90 | ± | 0.66 | −1.36 | 0.17 |
| IS | × | 245 |  |  | −0.80 | ± | 0.68 | −1.17 | 0.24 |
|  |  |  |  |  |  |  |  |  |  |
| Separation group × Sex | | | | |  |  |  |  |  |
| NS | × | Female |  |  | −0.23 | ± | 1.04 | −0.22 | 0.83 |
| CS | × | Female |  |  | −1.28 | ± | 0.72 | −1.77 | 0.08 |
| IS | × | Female |  |  | −1.52 | ± | 0.74 | −2.07 | **0.04** |
|  |  |  |  |  |  |  |  |  |  |
| PND × Sex | | | | |  |  |  |  |  |
| 50 | × | Female |  |  | −2.41 | ± | 0.64 | −3.75 | **<0.001** |
| 245 | × | Female |  |  | −1.39 | ± | 0.68 | −2.05 | **0.04** |
|  |  |  |  |  |  |  |  |  |  |
| Separation group × PND × Sex | | | | |  |  |  |  |  |
| NS | × | 50 | × | Female | 2.05 | ± | 1.30 | 1.58 | 0.11 |
| CS | × | 50 | × | Female | 2.34 | ± | 0.85 | 2.74 | **0.006** |
| IS | × | 50 | × | Female | 3.81 | ± | 0.87 | 4.36 | **<0.001** |
| NS | × | 245 | × | Female | 0.52 | ± | 1.18 | 0.44 | 0.66 |
| CS | × | 245 | × | Female | 1.05 | ± | 0.91 | 1.15 | 0.25 |
| IS | × | 245 | × | Female | 0.97 | ± | 0.97 | 1.00 | 0.32 |

**Table S10.** The effects of ELS and PND, and sex on (a) the total time spent freezing, (b) the total number of rearing, and (c) the total number of grooming behaviors demonstrated by the social housing (SH) and the IELS and DELS groups (no separation (NS), consecutive separation (CS), and intermittent separation (IS))

| Models | Variables | |  |  |  |  | Estimate | ± | SE | *z* | Pr(>\|*z*\|) |
| --- | --- | --- | --- | --- | --- | --- | --- | --- | --- | --- | --- |
| (a) Freezing behavior | | |  |  |  |  |  |  |  |  |  |
|  | Separation group | |  |  |  |  |  |  |  |  |  |
|  |  | NS, CS, IS |  |  |  |  | −0.34 | ± | 0.33 | −1.05 | 0.3 |
|  | PND | |  |  |  |  |  |  |  |  |  |
|  |  | 50 | |  |  |  | −0.43 | ± | 0.07 | −6.38 | **<0.001** |
|  |  | 245 | |  |  |  | 0.33 | ± | 0.06 | 5.73 | **<0.001** |
|  | Sex | |  |  |  |  |  |  |  |  |  |
|  |  | Female |  |  |  |  | −0.56 | ± | 0.29 | −1.93 | 0.054 |
|  |  |  |  |  |  |  |  |  |  |  |  |
|  | Separation group × PND | | | |  |  |  |  |  |  |  |
|  |  | NS, CS, IS | × | 50 |  |  | 0.16 | ± | 0.09 | 1.74 | 0.08 |
|  |  | NS, CS, IS | × | 245 |  |  | −0.65 | ± | 0.08 | −7.73 | **<0.001** |
|  |  |  |  |  |  |  |  |  |  |  |  |
|  | Separation group × Sex | | | |  |  |  |  |  |  |  |
|  |  | NS, CS, IS | × | Female |  |  | 0.64 | ± | 0.36 | 1.77 | 0.08 |
|  |  |  |  |  |  |  |  |  |  |  |  |
|  | PND × Sex | |  |  |  |  |  |  |  |  |  |
|  |  | 50 | × | Female |  |  | 0.84 | ± | 0.10 | 8.14 | **<0.001** |
|  |  | 245 | × | Female |  |  | 0.04 | ± | 0.10 | 0.41 | 0.68 |
|  |  |  |  |  |  |  |  |  |  |  |  |
|  | Separation group × PND × Sex | | | | |  |  |  |  |  |  |
|  |  | NS, CS, IS | × | 50 | × | Female | −0.97 | ± | 0.14 | −6.68 | **<0.001** |
|  |  | NS, CS, IS | × | 245 | × | Female | −0.29 | ± | 0.15 | −2.00 | **0.046** |
|  |  |  |  |  |  |  |  |  |  |  |  |
| (b) Rearing behavior | | |  |  |  |  |  |  |  |  |  |
|  | Separation group | |  |  |  |  |  |  |  |  |  |
|  |  | NS, CS, IS |  |  |  |  | 0.60 | ± | 0.28 | 2.12 | **0.03** |
|  | PND | |  |  |  |  |  |  |  |  |  |
|  |  | 50 | |  |  |  | 1.40 | ± | 0.15 | 9.10 | **<0.001** |
|  |  | 245 | |  |  |  | 0.02 | ± | 0.21 | 0.08 | 0.94 |
|  | Sex | |  |  |  |  |  |  |  |  |  |
|  |  | Female |  |  |  |  | 0.46 | ± | 0.26 | 1.80 | 0.07 |
|  |  |  |  |  |  |  |  |  |  |  |  |
|  | Separation group × PND | | | |  |  |  |  |  |  |  |
|  |  | NS, CS, IS | × | 50 |  |  | −0.62 | ± | 0.18 | −3.42 | **<0.001** |
|  |  | NS, CS, IS | × | 245 |  |  | 0.52 | ± | 0.24 | 2.19 | **0.03** |
|  |  |  |  |  |  |  |  |  |  |  |  |
|  | Separation group × Sex | | | |  |  |  |  |  |  |  |
|  |  | NS, CS, IS | × | Female |  |  | −0.28 | ± | 0.31 | −0.93 | 0.35 |
|  |  |  |  |  |  |  |  |  |  |  |  |
|  | PND × Sex | |  |  |  |  |  |  |  |  |  |
|  |  | 50 | × | Female |  |  | −0.58 | ± | 0.23 | −2.57 | **0.01** |
|  |  | 245 | × | Female |  |  | 0.60 | ± | 0.28 | 2.17 | **0.03** |
|  |  |  |  |  |  |  |  |  |  |  |  |
|  | Separation group × PND × Sex | | | | |  |  |  |  |  |  |
|  |  | NS, CS, IS | × | 50 | × | Female | 0.41 | ± | 0.27 | 1.54 | 0.12 |
|  |  | NS, CS, IS | × | 245 | × | Female | −0.77 | ± | 0.31 | −2.46 | **0.01** |
|  |  |  |  |  |  |  |  |  |  |  |  |
| (c) Grooming behavior | | |  |  |  |  |  |  |  |  |  |
|  | Separation group | |  |  |  |  |  |  |  |  |  |
|  |  | NS, CS, IS |  |  |  |  | 1.06 | ± | 0.61 | 1.73 | 0.08 |
|  | PND | |  |  |  |  |  |  |  |  |  |
|  |  | 50 |  |  |  |  | 1.20 | ± | 0.45 | 2.66 | **0.008** |
|  |  | 245 |  |  |  |  | 0.74 | ± | 0.54 | 1.38 | 0.17 |
|  | Sex | |  |  |  |  |  |  |  |  |  |
|  |  | Female |  |  |  |  | 1.44 | ± | 0.57 | 2.55 | **0.01** |
|  |  |  |  |  |  |  |  |  |  |  |  |
|  | Separation group × PND | | | |  |  |  |  |  |  |  |
|  |  | NS, CS, IS | × | 50 |  |  | −1.33 | ± | 0.53 | −2.53 | **0.01** |
|  |  | NS, CS, IS | × | 245 |  |  | −0.54 | ± | 0.59 | −0.91 | 0.36 |
|  |  |  |  |  |  |  |  |  |  |  |  |
|  | Separation group × Sex | | | |  |  |  |  |  |  |  |
|  |  | NS, CS, IS | × | Female |  |  | −1.19 | ± | 0.67 | −1.78 | 0.07 |
|  |  |  |  |  |  |  |  |  |  |  |  |
|  | PND × Sex | |  |  |  |  |  |  |  |  |  |
|  |  | 50 | × | Female |  |  | −2.41 | ± | 0.64 | −3.76 | **<0.001** |
|  |  | 245 | × | Female |  |  | −1.35 | ± | 0.68 | −1.98 | **0.048** |
|  |  |  |  |  |  |  |  |  |  |  |  |
|  | Separation group × PND × Sex | | | | |  |  |  |  |  |  |
|  |  | NS, CS, IS | × | 50 | × | Female | 2.98 | ± | 0.74 | 4.02 | **<0.001** |
|  |  | NS, CS, IS | × | 245 | × | Female | 0.99 | ± | 0.78 | 1.27 | 0.21 |
